# MulTI-domain self-management in older People wiTh OstEoarthritis and multi-morbidities: protocol for the TIPTOE randomised controlled trial

**DOI:** 10.1186/s13063-024-08380-7

**Published:** 2024-08-23

**Authors:** Rachel Deere, Philip Pallmann, Victoria Shepherd, Lucy Brookes-Howell, Andrew Carson-Stevens, Ffion Davies, Emma Dunphy, Preeti Gupta, Mary Hickson, Val Hill, Kate Ingarfield, Nicola Ivins, Fiona Jones, Robert Letchford, Rachel Lowe, Sarah Nash, Paula Otter, Hayley Prout, Elizabeth Randell, Bernadette Sewell, Debs Smith, Robert Trubey, Tom Wainwright, Monica Busse, Kate Button

**Affiliations:** 1 Centre for Trials Research, College of Biomedical and Life Sciences, Cardiff University, Cardiff, UK; 2 Division of Population Medicine, School of Medicine, Cardiff University, Cardiff, UK; 3 Healthcare NHS Foundation Trust is Homerton Hospital, Homerton Row, London, E9 6SR UK; 4 School of Health Professions, University of Plymouth, Plymouth, UK; 5 Faculty of Medicine, Health and Life Science, Swansea Centre for Health Economics, Swansea University, Swansea, UK; 6 School of Healthcare Sciences, Cardiff University, Cardiff, UK; 7 Population Health Research Institute, St George’s University, London, UK; 8 Bridges Self-Management, St George’s University, London, UK; 9 Orthopaedic Research Institute, Bournemouth University, Bournemouth, UK; 10 Public and Patient Involvement Member C/O Centre for Trials Research, Cardiff University, Cardiff, UK; 11 Cardiff and Vale University Health Board, Heath Park, Cardiff, CF14 4XN UK

**Keywords:** Osteoarthritis, Multiple long-term conditions, Self-management, Joint pain, Older adults

## Abstract

**Background:**

Four out of five people living with osteoarthritis (OA) also suffer with at least one other long-term health condition. The complex interaction between OA and multiple long-term conditions (MLTCs) can result in difficulties with self-care, restricted mobility, pain, anxiety, depression and reduced quality of life. The aim of the MulTI-domain Self-management in Older People wiTh OstEoarthritis and Multi-Morbidities (TIPTOE) trial is to evaluate the clinical and cost-effectiveness of the Living Well self-management support intervention, co-designed with people living with OA, integrated into usual care, in comparison to usual care alone.

**Methods:**

TIPTOE is a multi-centre, two-arm, individually randomised controlled trial where 824 individuals over 65 years old with knee and/or hip joint pain from their OA affected joint and at least one other long-term health condition will be randomised to receive either the Living Well Self-Management support intervention or usual care. Eligible participants can self-refer onto the trial via a website or be referred via NHS services across Wales and England. Those randomised to receive the Living Well support intervention will be offered up to six one-to-one coaching sessions with a TIPTOE-trained healthcare practitioner and a co-designed book. Participants will be encouraged to nominate a support person to assist them throughout the study. All participants will complete a series of self-reported outcome measures at baseline and 6- and 12-month follow-up. The primary outcome is symptoms and quality of life as assessed by the Musculoskeletal Health Questionnaire (MSK-HQ). Routine data will be used to evaluate health resource use. A mixed methods process evaluation will be conducted alongside the trial to inform future implementation should the TIPTOE intervention be found both clinically and cost-effective. An embedded ‘Study Within A Project’ (SWAP) will explore and address barriers to the inclusion of under-served patient groups (e.g. oldest old, low socioeconomic groups, ethnic groups).

**Discussion:**

TIPTOE will evaluate the clinical and cost-effectiveness of a co-designed, living well personalised self-management support intervention for older individuals with knee and/or hip OA and MLTCs. The trial has been designed to maximise inclusivity and access.

**Trial registration:**

ISRCTN 16024745. Registered on October 16, 2023.

**Supplementary Information:**

The online version contains supplementary material available at 10.1186/s13063-024-08380-7.

## Background

Osteoarthritis (OA) is a common long-term condition, with 85% of older adults presenting evidence of OA and more than 50% of people exhibiting radiographic evidence of OA in at least one joint by the age of 65 [1]. Knee, hip and small joints of the hand are the most commonly affected [2]. OA is associated with pain, swelling, muscle weakness, fatigue and the inability to do everyday tasks. Four out of five people living with OA suffer with at least one other long-term health condition [3], such as gastrointestinal or cardiovascular problems, frailty, clinically diagnosed depression, widespread pain and obesity [4, 5].

The complex interaction of painful OA and other conditions results in reduced independence and quality of life (QoL) in terms of self-care, mobility, usual activity, pain, anxiety and depression [6]. Individuals with OA and MLTCs are also less physically active than the general population, which has a negative impact on their physical and mental health [6, 7]. People living with MLTCs often struggle with the knowledge, confidence and skills to self-manage other conditions alongside their OA [8–10] can lead to high demand for coordinated interdisciplinary care, admission to hospital and long-term care, which is costly to the individual and society [11, 12].

Current NHS usual care for OA is condition-specific and does not typically consider the interplay between multiple conditions, or support a unified approach between services, healthcare practitioners, the individual with OA and MLTCs and their wider personal and social networks [13]. The needs and experiences of older people with OA and MLTCs have not been considered in the development of the current service model [14]. The National Institute for Health and Care Excellence (NICE) guidelines for knee and hip OA recommend core non-surgical and non-pharmacological treatments including (1) self-management education interventions, (2) exercise including strengthening and aerobic fitness, (3) weight loss if obese and (4) use of suitable footwear [15]. Updated guidance from the Osteoarthritis Research Society International in 2019 found that structured land-based exercise, yoga and weight management were effective and safe for all people with knee OA and MLTCs, but no core treatments were strongly recommended for people with hip OA and MLTCs [4]. These current clinical guidelines for OA are not underpinned by effectiveness studies conducted with older adults, people with MLTCs or individuals from under-served groups, such as those from areas of socioeconomic deprivation or ethnic minorities. Older people are frequently excluded from clinical trials due to the presence of MLTCs, difficulties accessing research opportunities, concerns around cognitive impairment and the ability to provide consent, and gatekeeping by health professionals and family members [16, 17]. Enhancing representation of under-served groups in clinical trials is important to ensure that research findings are widely applicable in practice [18].

The intervention trialled in TIPTOE study is a co-designed personalised living well self-management support intervention. It draws upon evidence from Bridges Self-management (Bridges) [19–21] and is informed by self-efficacy principles and social cognitive theory as the most successful foundation for self-management programmes [19, 22–25]. Bridges trained healthcare practitioners employ enabling language alongside referring to the co-designed Living Well book to support the development of self-management skills. In this way, people with knee and/or hip joint pain from their OA affected joint and at least one other long-term health condition are encouraged to access specific sources of self-efficacy, such as goal mastery and vicarious learning, to support knowledge, skills and confidence to self-manage whilst recognising the impact and interplay between their diet, activity and medications.

Social participation and community support are strongly associated with successful self-management [26] and TIPTOE will offer the opportunity for a nominated support person to join intervention sessions. Individuals with OA and MLTCs who have higher self-efficacy have been found to have a higher QoL [8, 9]. Throughout this paper, when we describe the ‘TIPTOE intervention’ we are referring to the aforementioned co-designed living well personalised self-management support intervention, which is detailed later in the methods.

## Methods

### Objectives

The objective of the TIPTOE randomised controlled trial is to evaluate the clinical and cost-effectiveness of adding the TIPTOE intervention to usual NHS care for people with knee and/or hip OA and MLTCs, compared to usual NHS care only. Intervention acceptability and feasibility will be measured alongside a detailed analysis of implementation enablers, barriers to adoption and sustainability beyond the trial timeline. An internal pilot starting from the beginning of recruitment and lasting 6 months will be used to determine whether the trial should continue as planned, continue with changes or be stopped completely. An embedded methodological study will focus on the recruitment and retention of traditionally under-served groups (e.g. ‘oldest old’, ethnic minorities, digitally excluded). The aim of this ‘Study Within A Project’ (SWAP) [27] is to identify barriers to the inclusion of people living with OA and MLTCs, with a focus on the intersection between people with MLTCs and other under-served groups. This will enable the trial to rapidly address the barriers through the development of tailored approaches to support recruitment and follow-up of these groups. Routine data will also be collected throughout the trial from NHS Digital (England) and the SAIL (Secure Anonymised Information Linkage) Databank (Wales), to determine the effect of the TIPTOE intervention on healthcare resource use.

### Study design and setting

**Fig. 1:**
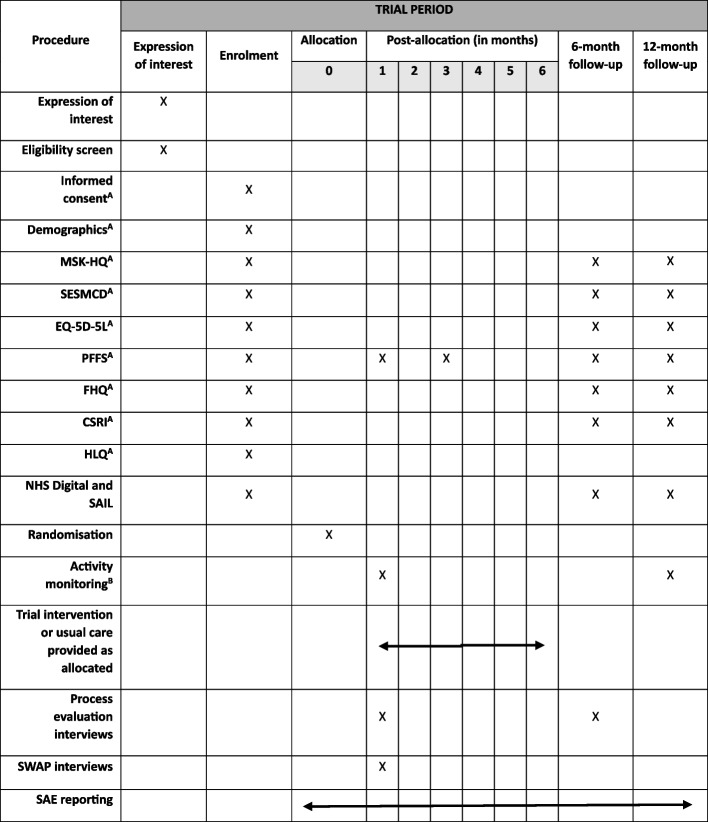
SPIRIT schedule of events. MSK-HQ, Musculoskeletal Health Questionnaire; SESMCD, Self-Efficacy Scale for Monitoring Chronic Disease; PFFS, Pictorial Fit-Frail Scale; FHQ, Falls History Questionnaire; CSRI, Client Service Receipt Inventory; HLQ, Health Literacy Questionnaire; SAIL, Secure Anonymised Information Linkage; SWAP, Study Within A Project; SAE, serious adverse event. ^A^Completed either online (with nominated support, if appropriate), or over the telephone with the central trial team. ^B^Attached on site by trial staff and returned in post or in person, or sent in post with instructions on how to attach and returned in post

This trial is an exploratory multi-centre, two-arm, individually randomised trial, with internal pilot and embedded mixed methods process evaluation, comparing the TIPTOE intervention integrated into usual care, to usual care alone for individuals with knee and/or hip OA and MLTCs. The schedule of events [28] is shown in Fig. 1.

Following informed consent and completion of baseline measures, 824 participants will be randomised using a minimisation process to either receive the TIPTOE intervention integrated into usual care or to continue to receive their usual care from the NHS for a period of 6 months. Recruitment is anticipated to last 17 months, with each participant taking part in the trial for approximately 13 months. Participants will be recruited from Wales and England through several methods including self-referral, being approached during routine clinic attendance, waiting list screening and invitation letters. Secondary care centres within England will be set up as individual sites and Wales will be set up as one site covering all seven Health Boards.

**Fig. 2:**
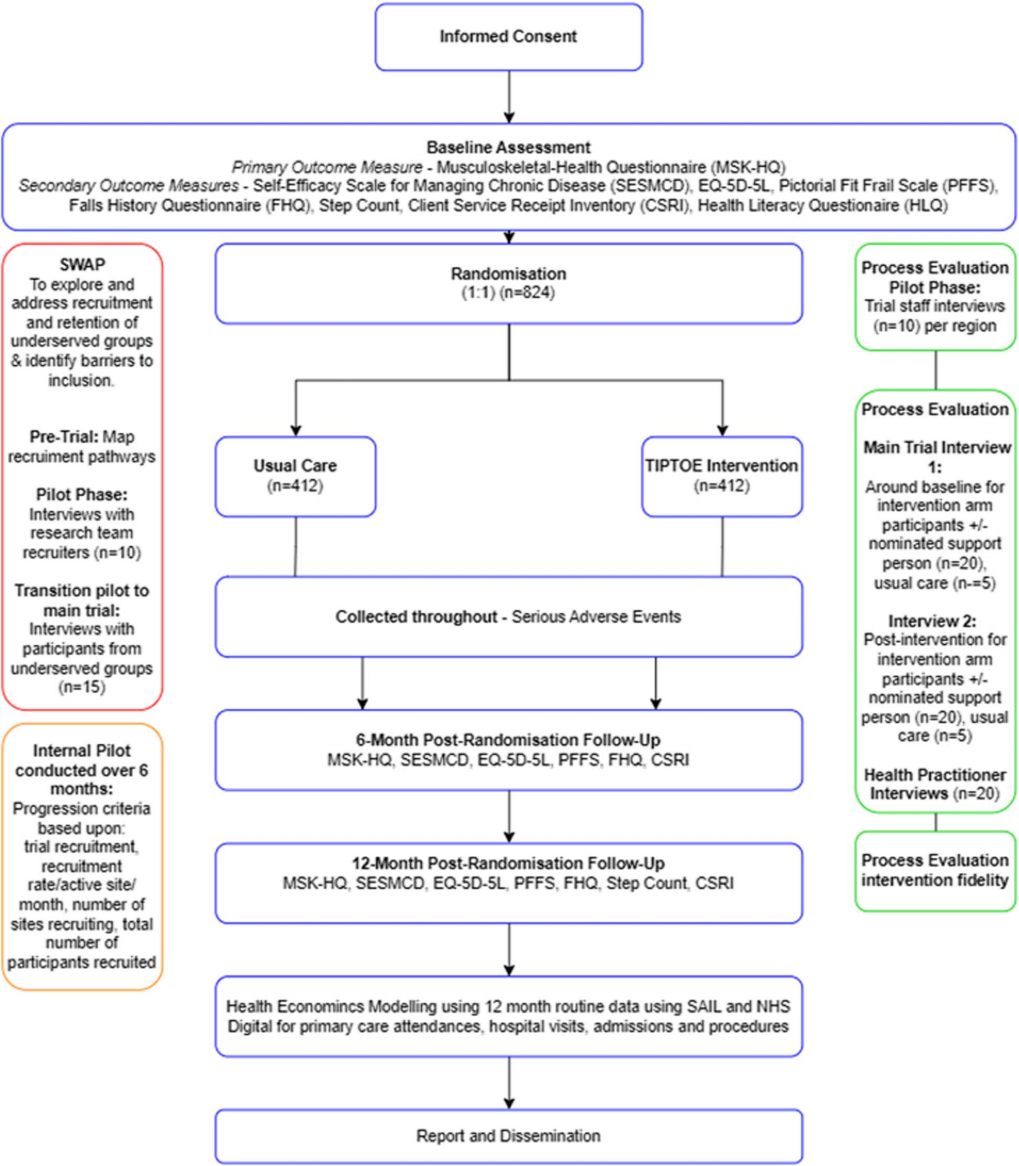
Participant flow diagram

Those who are eligible and consent to take part in the trial will complete a series of assessments at baseline with a selection of these repeated at 6 months and 12 months post-randomisation (Fig. 2). All consent and data collection will be primarily online, with telephone support provided where this is not possible or desirable from a participant perspective.

### Inclusion criteria

Eligible participants are adults aged 65 years and over, living independently in the community, or with carer support or in assisted living arrangements. They must have self-reported knee and/or hip joint pain in the OA affected joint which is impacting upon daily living and at least one other co-morbidity identified using the Self-Administered Co-morbidity questionnaire [29], or mild-to-moderate clinical frailty on the Canadian Study of Health and Ageing Clinical Frailty Scale [30] as determined by a healthcare practitioner.

### Exclusion criteria

Individuals will not be eligible to take part in TIPTOE if they have joint pain associated with a malignant condition, have undergone knee and/or hip joint surgery within the last 12 months on the affected joint, live in a care home (residential or nursing) or are unable to engage with the intervention based on clinician assessment.

## Interventions

### TIPTOE intervention

Participants allocated to the TIPTOE intervention arm will receive the personalised living well self-management support intervention integrated into their usual care. This will involve up to six one-to-one (lasting up to 60 min) personalised living well coaching sessions over 6 months with a trained healthcare practitioner. Participants will also receive a copy of the TIPTOE ‘Living Well’ book. Minimum engagement with the intervention is defined as having received a copy of the TIPTOE book and attended two sessions with the TIPTOE practitioner which is considered sufficient to facilitate application of the core intervention concepts.

The TIPTOE book has been co-designed with people living with joint pain and MLTCs and will be sent out to participants for use within and outside their one-to-one coaching sessions. This book includes stories and narratives of the experiences, challenges and successes of other people living with OA and MLTCs, which might act as a source of ideas or motivation. Through guided conversations participants will be encouraged to explore aspects of living with OA and MLTCs to identify what is important to them including small targets as well as hopes for the future and existing strengths and resources in relation to managing their conditions. Participants will be supported to set small daily targets based on their needs and preferences. At each session, participants will be encouraged to reflect on small successes and what they have learnt, to help them manage ups and downs in their health and build confidence in their ability to live well whilst navigating their health conditions.

**Fig. 3:**
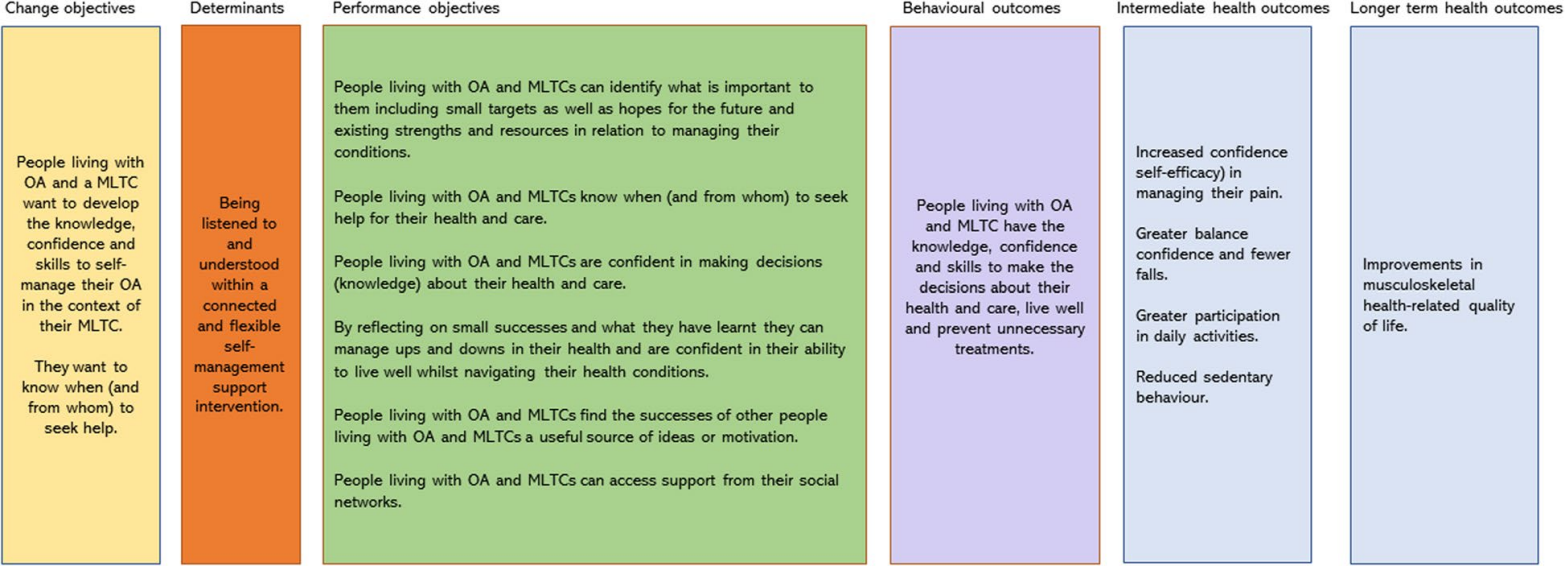
Logic model of change diagram for the TIPTOE intervention

The logic model of change diagram (Fig. 3) details the change objectives, determinants, performance objectives and proposed behavioural outcomes leading to intermediate and longer-term health outcomes for the person living with OA and a MLTC with the primary impact intended to be reflected by improvements in health-related QoL.

Practitioners will attend four 2-h training sessions provided by a Bridges Self-Management facilitator from a clinical background and co-delivered with an individual living with joint pain. Practitioners will be supported to develop their knowledge, skill and confidence in using nine key skills which are reflected in the TIPTOE intervention fidelity checklist. These nine key skills include strategies such as attentive listening, not rushing to fix, exploring how to use language to identify small things to focus on and reflect on successes, exploring wider social connections and then sustaining new approaches. These key skills have been developed through a combination of outputs from the co-designed Living Well with joint pain book and build on existing core principles used within Bridges training. Practitioners are encouraged and supported to integrate specific language and strategies which align with some or all of these core skills. Taken together, we hypothesise that a focus by practitioners using these core skills will enable participants’ (± their nominated supporter) confidence and knowledge to self-manage their everyday activities with joint pain and reduce the need for directive healthcare advice as the main source of learning. A programme of additional ongoing support to support practitioner intervention fidelity will include checklists, reminders, newsletter, exemplar videos and drop-in advice sessions. This support package will be available to all practitioners throughout the intervention delivery period. Attendance at drop-in sessions will also be monitored as well as topics for discussion, to build a bank of examples to share with the practitioner intervention delivery group.

Once practitioners have completed the training and fidelity checks (described below), they will be certified as Bridges trained TIPTOE practitioners.

### Fidelity of TIPTOE intervention delivery

To assess intervention fidelity, healthcare practitioners will complete a pre-post training questionnaire addressing their knowledge, skills and confidence on a 3-point Likert scale covering a range of items relating to the key principles of Bridges Self-management and contextualised to the delivery of the TIPTOE intervention. Additionally, healthcare practitioners will be asked to audio record three coaching sessions, which will be evaluated against an adapted pre-existing fidelity checklist for the key attributes they are expected to demonstrate during intervention delivery [31]. Participants will be made aware if their session will need to be recorded, and each recording will be stored in a secure space only accessible by the trial team and TIPTOE practitioner. Reflections from the practitioners delivering the intervention will be captured through online notes pages, completed after each participant session, only accessible by the TIPTOE practitioner and the central trial team.

### Usual care

All participants randomised to the control arm will continue to receive their usual care as currently available in the NHS within the participant’s region and services. Usual care for OA typically involves pathology specific assessments and treatment services with individuals with OA being offered a combination of core treatments such as exercise, weight management, self-management education and advice. This may be on an individual or group basis in primary, secondary or community care. We will record the usual care received by completing a bespoke Client Services Receipt Inventory (CSRI) at baseline and 6 and 12 months post-randomisation, in both arms. We will also focus on exploring usual care as part of the mixed methods process evaluation.

Participants in the control group will receive the co-designed Living Well book following completion of their 12-month follow-up.

### Participation changes

Participants have the right to change their level of participation in the trial at any time, without it affecting their ongoing care. If a participant initially consents but subsequently wishes to change their level of participation, clear distinctions will be made as to what aspect of the trial the participant wants to change. These include (1) withdrawal from the intervention only; (2) partial withdrawal from further data collection; (3) complete withdrawal from further data collection, incl. data linkage; and (4) withdrawal of permission to use data already collected.

## Outcomes

### Primary outcome

The primary outcome measures are symptoms and QoL, assessed on the Musculoskeletal Health Questionnaire (MSK-HQ). The MSK-HQ is a 14-item scale which measures the holistic effect of MSK pain on symptoms and health-related QoL. The MSK-HQ has been shown to be both valid and reliable [32, 33]. It will be measured at baseline, 6 months (end of intervention) and 12 months (to assess in a repeated measures analysis if the intervention has a sustained effect).

### Secondary outcomes

All secondary outcome measures are listed below and will also be assessed at baseline, 6 and 12 months, unless otherwise stated.

i. The Self-Efficacy Scale for Managing Chronic Diseases (SESMCD) [34] a 6-item scale, measuring 3 types of self-efficacy beliefs: performing specific behaviours, general disease management and achievement outcomes.
ii. Directly measured step count, collected with an ActivPAL physical activity monitor. Physical activity monitoring will be completed at baseline and 12-month follow-up only.
iii. The EQ-5D-5L [35] consisting of 5 descriptive dimensions (mobility, self-care, usual activities, pain/discomfort and anxiety/depression) and a visual analogue scale (VAS) of participants’ self-rated health.
iv. The Pictorial Fit-Frail Scale (PFFS) [36, 37] a 14-domain self-assessment of frailty. Levels of ability are represented with images.
v. The Falls History Questionnaire (FHQ) [38] combining falls history and falls self-efficacy.
vi. Healthcare resource use—obtained through consented linkage to NHS Digital and SAIL. Datasets will include primary care (where available), hospital admissions, surgery, outpatient and emergency care. A CSRI will also be used to capture additional healthcare resource use, which cannot be obtained through NHS Digital or SAIL in the detail required (e.g. primary care data, social care data, prescription data). The CSRI is adapted specifically to capture the health care resource use of patients with OA.
vii. The Health Literacy Questionnaire (HLQ) [39] to evaluate the cognitive and social skills which determine the motivation and ability of individuals to gain access to, understand and use information in a way that promotes and maintains good health. The HLQ will only be administered at baseline.

### Sample size

The TIPTOE trial aims to recruit a total of 824 individuals with knee and/or hip OA and MLTCs. We aim to detect a minimum clinically important difference (MCID) of 0.3 standard deviations (SDs) between the randomised arms in the primary outcome of the MSK-HQ with 90% power whilst controlling the two-sided type 1 error level at 5%. This effect size is consistent with previous research where MCIDs of 0.3 SDs or greater were established for different pain sites [33]. An individually randomised trial with a normally distributed endpoint measured at a single follow-up time point would require 470 participants. Power in repeated measures designs, however, depends on additional factors such as the correlation between repeated measurements [40]: with non-zero correlation the power of a test for mean treatment difference between arms across follow-up time points in a mixed-effects regression model for repeated measures will exceed the power of a two-sample design if the effect size remains the same (0.3 SDs) across follow-up. The latter is unlikely in this study where the difference between arms might ‘peak’ around the time the intervention programme ends and not be fully sustained at 12 months, or conversely, where the full effect might not yet have become manifested at 6 months. Using GLIMMPSE (University of Colorado, Denver, USA) version 3.0.0 with calculations based on the Hotelling-Lawley trace test, which is equivalent to a mixed-model test [41], we studied the power of designs with *n* = 470 under a range of plausible scenarios involving first-order autoregressive correlation structures over time with parameter rho between 0.3 and 0.7 and treatment effects at least the size of the MCID at least at one follow-up time point whilst assuming no difference between treatment arms at baseline due to randomisation. The power exceeded 90% for most of these scenarios and never fell below 83.5%. For most scenarios, between 70 and 90% power was also achieved for testing differences in trend over time between arms.

The delivery of the intervention across an expected 8 sites by up to 16 healthcare teams may induce additional clustering in the intervention arm, leading to an inflation of the above sample size. Based upon Moerbeek and Wong’s formula [42, 43], with an intra-cluster correlation coefficient (ICC) of 0.02 in the intervention arm, *n* = 576 will be required for 90% power in a balanced design using a 1:1 randomisation ratio, corresponding to 288 in the control group, and an average of 18 per healthcare team in the intervention arm. Expecting 30% loss to follow-up based upon published dropout rates for trials of lifestyle interventions for multi-morbidity [44, 45], a total of 824 individuals will be recruited.

### Recruitment

Potential participants may be identified by the following methods:

1. Waiting lists—individuals on NHS waiting lists (e.g. trauma and orthopaedic, or physiotherapy outpatients) will be screened by site staff and an invitation sent via email, letter or text message from the trial site staff.
2. Routine clinic attendance—individuals attending routine clinic appointments at trial sites will be screened for eligibility during routine appointments with site staff.
3. Screening GP databases—GP surgeries set up as Participant Identification Centres (PICs) will screen their databases according to the eligibility criteria and send out invitations via letter or text to potentially eligible participants.
4. Self-referral—advertisements will be provided to trial sites, GP surgeries, community centres, pharmacies and health centres so that they can advertise the trial on their premises and during clinics or support groups. We will also advertise the trial on social media.

All outward-facing communications (including audio and filmed materials) about the project will be reviewed by the public involvement advisory group for representativeness, accessibility and inclusivity.

### Data collection

Consent and all study data will be collected electronically using a secure online system, the ‘ASSISTANT’ system (https://studyassistant.org.uk/). It combines a secure purpose-built study website database designed by Baseline Software Ltd (East Sussex, UK) (https://TIPTOE.org.uk/) and utilises REDCap [46, 47], hosted by Cardiff University, as a data repository. All data entered by participants (or the trial team when support is required) are thus stored confidentially, only accessible to the central trial team and allocated site staff. The study website has built-in validations to promote data quality, e.g. double data entry, range checks, missing data, etc. which have been extensively tested.

### Expression of interest and consent

Potential participants identified by TIPTOE sites or through responding to advertisements will be directed to the TIPTOE website (see ‘Data collection’ section for overview of website). The TIPTOE website contains summary, pictorial and detailed Participant Information Sheets (PIS). Interested individuals are invited to complete the expression of interest (EOI) form within the website. This form will collect individuals’ name, age, contact details and eligibility information. The website will automatically determine whether potential participants are eligible. If there are any queries, the central trial team may call potential participants to check eligibility. Personalised emails will be sent in response to all EOIs to thank them for their interest and inform them, based on the information they have provided, whether they are eligible to take part in the trial or not. Eligible participants who can be allocated to a TIPTOE research site will be sent a secure link via email to complete the consent form.

If those interested in taking part are unable or unwilling to use the study website, local advertisements will contain a central telephone number to contact the central trial team, who will provide support in completing the EOI and consent forms. Alternatively, potential participants identified by a TIPTOE site can consent in person with a delegated member of the team at the site. Potential participants are also encouraged to join the trial with a nominated supporter (e.g. friend, family member or carer) who can support them in using the study website.

Following initial provision of informed consent, participants with MLTCs such as dementia may experience a decline in their cognitive function during participation. Where there are concerns about a participants ability to provide ongoing consent, capacity will be assessed by an appropriately trained member of the research team in accordance with the Mental Capacity Act 2005 [48]. If required, a personal consultee will be approached to provide advice about their continued participation [48]. This will usually be a family member or close friend and may be the person acting as the nominated supporter. Where a personal consultee is not available, a nominated consultee may be identified and approached. They will be provided with a consultee information sheet and asked for their advice about whether the participant would want to continue in the trial. If the consultee advises that the participant would wish to continue in the trial, they will be asked to complete a consultee declaration form; otherwise, the participant will be withdrawn.

### Baseline assessments

Once participants have provided consent to take part in the trial, they will be asked to complete baseline questionnaires on the website (see Fig. 1 and ‘Primary outcome’ and ‘Secondary outcomes’ sections for details). An email link will take participants directly to the questionnaires and their responses will be automatically saved to the TIPTOE REDCap database repository. For participants who are unable or unwilling to complete the baseline measures on the trial website, the central trial team will contact via telephone to complete the baseline measures with the participant verbally.

### Randomisation method and implementation

Following completion of the baseline measures, participants will be individually allocated in a 1:1 ratio to the TIPTOE intervention or usual care arm using a minimisation algorithm with a random element [49]. Minimisation is based upon self-reported gender (male, female or other), OA site (knee, hip or both) and trial site. Participants and allocated sites will receive email notification informing them of which arm the participant has been randomised to.

### Follow-up

At 6 and 12 months post-randomisation, all participants will receive an email reminder requesting them to complete a selection of the questionnaires they completed at baseline (see Fig. 2). All participants who have completed both their 6- and 12-month follow-ups will be eligible to be entered into a prize draw for vouchers worth £100 with a 1 in 20 chance of receiving the incentive. There will be 2 draws.

### Activity monitoring

At baseline and 12-month follow-up, participants will be asked to wear an activity monitor (ActivPAL) on their thigh for 7 days and 7 nights to obtain an assessment of their physical activity. The ActivPAL4 device provides measures for a range of metrics including step count and time spent lying, sitting and standing. The device is covered in a nitrile sleeve and attached to the front of the participant’s thigh using a piece of Tegaderm®. The device will be posted to the participant by the central trial team. A demonstration video and instructions will be accessible on the TIPTOE website, explaining how to fit the device, what to do if they have any problems with the device and how to contact the trial team if support is required. Following the 7 days and nights, participants will be asked to return the device to the central trial team by posting via pre-arranged services. Data will be downloaded by the central trial team using the ActivPAL docking station and ActivPAL software and saved on a secure research drive. No identifiable data will be stored or collected by the ActivPAL devices.

## Process evaluation

In accordance with Medical Research Council guidelines for the development and evaluation of complex interventions [50, 51], an embedded mixed methods process evaluation will be conducted to explore trial process, intervention mechanisms and context, to inform future implementation of the TIPTOE intervention. The mixed methods process evaluation will be performed across three phases.

During the pilot phase of the trial, site staff from each region will be invited to take part in semi-structured interviews remotely. The aim of these interviews is to evaluate training and trial processes and potential sources for contamination between the intervention and control arms in trial delivery. Thematic analysis and rapid qualitative review will be used to feedback to the trial team and trial management group (TMG) to inform the main trial.

During the main phase of the trial, semi-structured interviews will be conducted with a subset of participants receiving the TIPTOE intervention (*n* =  ~ 20) and those receiving usual care (*n* =  ~ 5). Participants will be interviewed at baseline and post-intervention. We will aim to interview the same participants at both time points where possible. We will seek additional participants post-intervention, if it is not possible to interview the same participants from baseline. We will do this through sending out email reminders and highlighting the need for more interviewees on the monthly newsletter. Baseline interviews with participants receiving the TIPTOE intervention will aim to capture descriptions of usual care, contextual background (i.e. health experiences) and issues around the acceptability of the intervention (i.e. early perceptions, concerns, etc.). Baseline interviews with participants in the usual care group will also aim to capture descriptions of usual care as well as their experiences of trial processes (i.e. randomisation, recruitment, etc.). Post-intervention interviews with participants who received the TIPTOE intervention will aim to explore the acceptability of the intervention, adherence (did patients adhere to the intervention, did they alter it at all), advantages and disadvantages to the intervention, and barriers and facilitators to the intervention. Post-intervention interviews with the usual care participants will aim to explore if there have been any changes to their usual care and health experiences.

During the main phase of the trial, semi-structured interviews will also be conducted with healthcare practitioners (*n* =  ~ 20) who have been delivering the TIPTOE intervention. The interview guide will be based on domains from the updated Consolidated Framework for Implementation Research which acknowledges complex interactions between constructs that are associated with effective implementation [52]. We will purposefully sample for maximum variation across regions and across professional background (e.g. occupational therapists, physiotherapists, podiatrists, dietitians, pharmacists, nurses). The interviews will explore professionals’ views on acceptability (their views on the advantages and disadvantages of the intervention, barriers and facilitators to implementing the intervention, their perceptions on patients’ views on the intervention), adherence (their perceptions of whether patients adhered to the intervention, whether they made local changes) and adaptation (did the intervention work for this population group, did the healthcare practitioner have experience of using Bridges before the trial), reflections on training needs (was the training sufficient, how could it be improved) as well as trial processes (recruitment, randomisation, consent processes, patient materials, communication with the trial team).

Each healthcare practitioner delivering the intervention will be observed according to pre-determined quality markers which have been derived from previous research and co-design stages. They will be required to send in an audio or filmed recording of three of their intervention sessions. Feedback on their use of language which aligns with fidelity criteria will be provided by the Bridges team (who will have delivered training).

### Internal pilot

An internal pilot will be carried out from the start of recruitment and assessed after 6 months using a traffic light system [53, 54]. Green will result in the trial continuing as planned, amber will result in the trial continuing with changes, and red will result in the trial stopping.

### Study Within A Project (SWAP)

The SWAP will build on existing knowledge around the inclusion of older people [16, 42], people with impaired capacity to consent [17] and other under-served groups [18]. A mixed methods approach will be used to explore the methodological and systemic barriers to including older people with MLTCs in the trial through three phases.

During the pre-trial phase, we will establish the characteristics of the clinical population(s). Using a process mapping approach [55], we will work collaboratively with sites to map recruitment pathways to local care pathways with a focus on barriers to the inclusion of groups who are identified as under-served in this context, e.g. people with MLTCs, ‘oldest old’ and those with cognitive impairment, and other intersecting factors such as people from ethnic minority groups, people living in remote areas and those who are socioeconomically disadvantaged.

During the pilot phase of the trial, we will explore the attitudes, skills and confidence of research team members in recruiting participants from populations that are under-served through a series of one-to-one interviews (*n* =  ~ 10).

From this, we will transition from the pilot into the main phase of the trial using the SEAR (Screened, Eligible, Approached, Randomised) framework which aims to improve the process of recruitment to randomised controlled trials [56]. We will examine the proportion of people from under-served groups who were screened, identified as eligible and recruited to the trial, together with follow-up and retention rates. We will carry out one-to-one interviews with participants from under-served groups and their carers or supporters (*n* =  ~ 15, 10 from intervention arm and 5 from usual care arm) to explore their experiences of participating in the trial. Participants will be purposively sampled to reflect diversity, such as those population characteristics noted in the pre-trial mapping phase. This may include situations where a participant’s cognitive function affected their ability to engage (or remain engaged) with the intervention, and the feasibility of increasing support or providing an alternative option such as a supported form of self-management (a version of which is currently under development by the research team as ‘SUSTAIN’) [57].

Transcripts will be analysed through inductive thematic analysis informed by the findings from the previous phase. We will use a systems-based approach [58] to triangulate the findings with previous phases to identify barriers to the inclusion of people from under-served populations at either an individual, recruiter/site or trial level. Facilitative strategies will then be designed for rapid implementation during the main trial.

### Statistical analysis

Participant characteristics and baseline data will be summarised descriptively by allocation (intervention or usual care). Data analysts will be blinded to treatment group. The primary analysis will be intention-to-treat (i.e. all randomised participants, regardless of level of adherence to the allocated intervention or ‘contamination’ of usual care, with complete or incomplete follow-up, will be included), and use a partially clustered multi-level model, i.e. a linear mixed-effects regression model with an unstructured covariance structure imposed on the residuals to model the covariance between repeated measures on the same participant [59], and random cluster effects in the intervention arm to account for groups of participants receiving the intervention from the same healthcare team, allowing for heteroskedastic individual-level errors [60]. The model will include categorical time, treatment and their interaction as fixed effects, as well as any factors used to balance the randomisation in the minimisation algorithm. Baseline MSK-HQ will be included in the response variable. This model will be used to compare the mean MSK-HQ scores across follow-up time points between participants receiving the TIPTOE intervention and those receiving usual care using appropriate linear contrasts and a Kenward-Roger approximation to the denominator degrees of freedom [61]. Results will be presented as point estimates alongside two-sided 95% confidence intervals (CIs) and *p* values, as well as variance components and ICCs for healthcare team clustering. Clinical effectiveness will be concluded if the point estimate of the treatment effect favours the intervention, and the 95% CI excludes zero. Similar analyses will be performed for the secondary outcomes.

In secondary analyses, we will estimate the treatment effect at 6- and 12-month follow-up separately based upon the primary analysis model. We will also assess a potential difference in trend over time between the two arms. The primary analysis will be repeated with a random coefficients model using random effects for each participant’s intercept (and possibly slope) instead of the covariance pattern model described above. A per-protocol or complier average causal effect (CACE) analysis will explore the effect of treatment adherence whilst maintaining groups as randomised. Multiple imputation will be considered as a sensitivity analysis if warranted by the proportion of missing follow-up data. Other sensitivity analysis, such as modelling the outcome variable at baseline as a fixed-effects covariate and fitting a residual correlation structure that is stratified by treatment arm, will be considered. Planned subgroup analyses will involve stratification by (1) type of usual care provided at sites, (2) level of fidelity to the intervention, (3) mode of completion of outcome measures (self-reported versus proxy) and (4) number and type of long-term conditions. Safety outcomes will be tabulated by arm and analysed using logistic regression as appropriate. A comprehensive statistical analysis plan (SAP) will be agreed and signed off prior to database lock. Findings will be reported according to the Consolidated Standards of Reporting Trials (CONSORT) statement [62].

### Qualitative analysis

Audio-recorded remote (telephone or video-conference) interviews will be transcribed verbatim using a professional transcription service and de-identified. Transcripts will be analysed using an inductive thematic approach and will consider the different interviewee characteristics, e.g. the different sites. Qualitative data will be managed using the qualitative software package NVivo to enable initial coding and categorisation of raw data. A thematic framework will be developed based on the research objectives and emerging themes. A proportion of transcripts will be double-coded by an additional researcher, and discrepancies discussed until consensus is reached. A qualitative data collection and analysis plan (QAP) will be developed to detail how the analysis will be carried out.

A triangulation exercise will be carried out to combine quantitative results on fidelity, intervention implementation and usual care with the qualitative data analysis results, using an adapted triangulation protocol technique [63, 64]. Triangulation will allow for an in-depth understanding of the functioning of the intervention, mechanisms and contextual factors and the implementation process to support TIPTOE beyond this trial. The data will support any required refinement of the logic model of change.

### Health economic evaluation

We will carry out a within-trial and lifetime cost-utility analysis (CUA) as well as a cost-consequences analysis comparing the relative costs and outcomes of the TIPTOE intervention with usual care. The within-trial CUA will use the EQ-5D-5L scores from baseline and 6- and 12-month follow-up to calculate the number of quality-adjusted life years (QALYs) gained (or lost) as a result of the intervention using an area-under-the-curve approach. Furthermore, we will collect participants’ healthcare resource use using a CSRI specifically adapted to individuals with OA and MLTCs. Intervention implementation cost will be collected as part of the process evaluation and through discussions with the trial team and will include intervention adaptation and provision, administration, and staff costs for training and delivery. All-cause healthcare resource use (including outpatient visits, inpatient stays and emergency care) will be obtained from NHS Digital and SAIL. Published unit costs will be used to value healthcare resource use at the most current price year. The base case analysis will use the intention-to-treat and linear mixed-effects regression model for repeated measures commensurate with the statistical analysis. Costs and utilities will be presented as point estimates alongside two-sided 95% CIs and *p* values, as well as incremental costs of the intervention compared to usual care. The incremental cost-effectiveness ratio (ICER) of the intervention (expressed as cost in £ per QALY gained) will then be calculated at 12 months from a UK NHS and personal social services (PSS) perspective using the intention-to-treat population as the base case and multiple imputation commensurate with the statistical analysis as sensitivity analysis. Following the within-trial evaluation, a decision analytic model will use the 6- and 12-month healthcare resource use data to extrapolate the longer-term (between 12 months and lifetime) cost-effectiveness of the intervention from a UK NHS and PSS perspective, expressed as cost per QALY gained. We will perform structured literature searches to provide data inputs that cannot be obtained from trial data. A health economic analysis plan (HEAP) will be agreed and signed off prior to database lock. Findings will be reported according to the Consolidated Health Economic Evaluation Reporting Standards (CHEERS) statement [65].

### Oversight and monitoring

A trial management group (TMG) will meet bi-monthly to review trial progress and recruitment targets. There will also be an external Trial Steering Committee (TSC) providing independent oversight and an Independent Data Monitoring Committee (IDMC) to review participant safety. All members of these committees have signed the respective trial specific charters. The TIPTOE trial team agree to allow trial related monitoring, including audits and regulatory inspections, by providing direct access to source data as required. Participant consent for this will also be obtained as part of the informed consent process.

### Safety

Participants will be asked to use the ‘report a problem’ link on the study website, or to inform their local healthcare professional to report any relevant medical events that occur during their time on the trial (from giving consent to 12-month follow-up). In this patient population, high rates of acute illness resulting in hospitalisation, new medical problems, deterioration of existing medical problems and death are expected. This information will be collected as part of the routine follow-up and will not be subject to serious adverse event (SAE) reporting. We ask for participants to report if they have a fall that results in hospitalisation or an SAE thought to be related to the trial intervention or research procedures. An SAE is defined as any adverse event that (1) results in death, (2) is life threatening, (3) requires hospitalisation or (4) results in persistent or significant disability or incapacity. The site principal investigator will review the event to determine if it meets the trial’s definition of an SAE. If so, an SAE form will be completed by the site team. All safety reports will be reviewed by the central trial team for completeness and uploaded to the trial database.

### Roles and responsibilities

As sponsor, Cardiff University has delegated all trial responsibilities to the Centre for Trials Research (CTR) at Cardiff University. Dr Kate Button and Professor Monica Busse are co-chief investigators and conceptualised the TIPTOE trial with Bridges Self-management, alongside co-applicants from Cardiff University, St George’s University of London, Swansea University, Bournemouth University, the University of Plymouth, Cardiff and Vale University Health Board, Homerton NHS Foundation Trust and a patient and public involvement and engagement (PPIE) representative. All trial activities will adhere to the CTR’s standard operating procedures (SOPs).

## Discussion

Knee and/or hip OA affects 1 in 2 older adults, with nearly 7 in 10 OA sufferers having more than one other medical condition. This trial will examine the clinical and cost-effectiveness of the TIPTOE intervention across multiple sites and services in Wales and England. Routine data will be collected throughout the trial to inform healthcare resource use. For participants in England, we will work with NHS Digital to obtain prospective linked Hospital Episode Statistics (HES) data, emergency care data and GP data. For participant in Wales, we will seek to obtain the equivalent data from the Patient Episode Dataset for Wales (PEDW), Outpatient Dataset for Wales (ODW), Emergency Department Dataset Daily (EDDD) and the Welsh Longitudinal GP Dataset (WLGPD). The embedded process evaluation will inform subsequent implementation should the intervention be shown to be clinically and cost-effective.

The SWAP will inform the trial to ensure for maximum inclusivity, whilst the provision of multiple routes of participant identification, various modes of information delivery (written and visual), data collection (online or via telephone) and intervention delivery (face-to-face or video conferencing) from the outset also aims to maximise inclusion. Wales will form a single site under a ‘One Wales’ model facilitating recruitment across Wales regardless of location, an approach which has been successfully implemented previously [66]. Sites in the South East and South West of England, London, and the North will also be opened to recruitment.

Extensive patient and public involvement and engagement (PPIE) input has been employed in the project, following the UK Standards for Public Involvement [67], from the co-design of the TIPTOE intervention through to the development of the trial protocol and patient facing documents and materials. Additionally, the health practitioner training which is overseen by Bridges Self-Management will be co-delivered with an individual living with joint pain. Our PPIE members will continue to support the trial team with ongoing advice and input into recruitment strategies, advertisements and later dissemination planning. Two PPIE members are part of the trial management group. We are utilising the Public Involvement in Research Impact Tool to encourage discussion, collaboration and a transparent approach to public involvement planning and impact reporting in TIPTOE.

The TIPTOE trial will form an evidence base for whether services should integrate this self-management intervention, and the benefits and costs of doing so.

## Study status

This manuscript has been drafted according to version V4.1 (15/07/2024) of the trial protocol and in accordance with the SPIRIT checklist. The trial opened to recruitment in January 2024, with recruitment anticipated to end by March 2025.

### Supplementary Information

Supplementary Material 1

## Data Availability

All trial data will be stored on the TIPTOE Research Data Storage space in a secure repository held by Cardiff University. The CTR is a signatory of AllTrials and aims to make its research data available wherever possible. Data requests will undergo a review process to ensure that the proposal complies with patient confidentiality, regulatory and ethical approvals and any terms and conditions associated with the data. On study completion, trial data will be made available upon reasonable request and following CTR data sharing policies. Requests to access the data should be made to the ctrdatasamplerequests@cardiff.ac.uk. The datasets analysed during the current study and statistical code are available from the corresponding author on reasonable request, as is the full protocol.

## Abbreviations

CACE: Complex average causal effect
CHEERS: Consolidated Health Economic Evaluation Reporting Standards
CI: Confidence intervals
CONSORT: Consolidated Standards of Reporting Trials
CSRI: Client Service Receipt Inventory
CTR: Centre for Trials Research
CUA: Cost utility analysis
EDDD: Emergency Department Dataset Daily
EOI: Expression of interest
FHQ: Falls History Questionnaire
HEAP: Health Economics Analysis Plan
HES: Hospital Episode Statistics
HLQ: Health Literacy Questionnaire
ICC: Intra-cluster correlation
ICER: Incremental Cost-Effectiveness Ratio
IDMC: Independent Data Monitoring Committee
MCID: Minimal clinically important difference
MLTCs: Multiple long-term conditions
MSK-HQ: Musculoskeletal Health Questionnaire
NICE: The National Institute for Health and Care Excellence
OA: Osteoarthritis
ODW: Outpatient Dataset for Wales
PEDW: Patient Episode Dataset for Wales
PFFS: Pictorial Fit-Frail Scale
PICs: Patient Identification Centres
PIS: Participant Information Sheet
PPIE: Patient and public involvement and engagement
PSS: Personal social services
QALYS: Quality-adjusted life years
QAP: Qualitative analysis plan
QoL: Quality of life
SAE: Serious adverse event
SAIL: Secure Anonymised Information Linkage
SAP: Statistical analysis plan
SDs: Standard deviations
SEAR: Screened, Eligible, Approached, Randomised
SESMCD: Self-Efficacy Scale for Managing Chronic Diseases
SOPs: Standard operating procedures
SWAP: Study Within A Project
TMG: Trial management group
TSC: Trial Steering Committee
WLGPD: Welsh Longitudinal GP Dataset
